# How HIV Sneaks aboard Mature Dendritic Cells

**DOI:** 10.1371/journal.pbio.1001454

**Published:** 2012-12-18

**Authors:** Caitlin Sedwick

**Affiliations:** Freelance Science Writer, San Diego, California, United States of America

**Figure:**
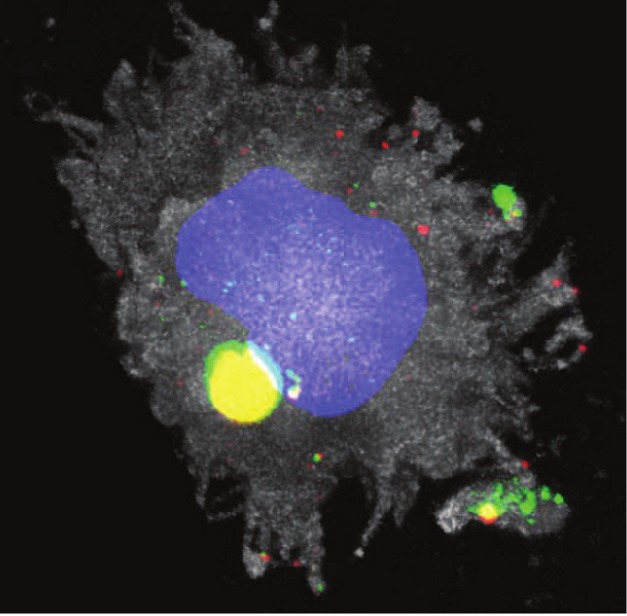
The receptor Siglec-1 (green) captures and retains HIV (red) within the cytoplasm of a mature dendritic cell (grey). Both HIV and Siglec-1 accumulate in a sac-like compartment (yellow) near the dendritic cell nucleus (blue). Image credit: Maria Pino, Itziar Erkizia, and Nuria Izquierdo-Useros.

HIV, the virus that causes AIDS, has an extensive repertoire of tricks that it uses to evade, manipulate, and subvert the human immune system. Perhaps most insidious is the virus's ability to turn the immune system's defensive tactics to its own advantage. For example, HIV-1 (the virus that causes most cases of HIV infection) uses a type of immune cell, the dendritic cell (DC), to spread its infection.

DCs found in most peripheral tissues are in an immature state. However, those found in lymphoid tissues are more highly differentiated, or mature, and help trigger protective immune responses by interacting with another kind of immune cell, the T cell. Unfortunately, T cells happen to be HIV's favorite host cell, and mature DCs can acquire HIV particles from HIV-infected T cells. These viruses are swallowed and then stashed in a sac-like compartment within DCs, where they wait to assault new T cells.

Earlier this year, a collaboration between two groups, led by Nuria Izquierdo-Useros and Javier Martinez-Picado in Spain, and Maier Lorizate and Hans-Georg Kräusslich in Germany, examined how mature DCs recognize and take up HIV virions. They showed that mature DCs recognize sialic acid–containing glycolipids (termed gangliosides) that are present in the HIV's lipid envelope. But at that time it still wasn't clear which receptor on the DC surface was used to recognize these glycolipids. Now, in this month's issue of *PLOS Biology*, Izquierdo-Useros and colleagues return with the answer to this question.

Work by other groups had suggested that a surface protein called DC-SIGN might be the DC receptor for HIV. But DC-SIGN interacts with viral glycoproteins and not with membrane glycolipids. Moreover, HIV capture by DCs is strongly enhanced when DCs mature in the presence of pro-inflammatory compounds such as lipopolysaccharide (LPS). As LPS is often present at high levels during HIV infection, it's a good bet that many DCs have reached their mature state under its influence. And yet, DC-SIGN expression levels do not significantly change after DCs are exposed to LPS, so DC-SIGN cannot be responsible for enhanced viral uptake after DC maturation with LPS. The authors therefore argued that a different DC surface receptor is probably involved.

To identify other DC proteins responsible for HIV uptake, the authors first examined how surface protein expression patterns change after LPS maturation of DCs. They focused their analysis on Siglecs, a family of proteins that are known to bind to sialic acid–containing molecules, including membrane gangliosides. Indeed, they found that one Siglec in particular, namely Siglec-1, is strongly upregulated in LPS-stimulated DCs.

This finding prompted the researchers to test whether Siglec-1 contributes to HIV uptake by DCs. They observed that increased Siglec-1 expression on the surface of DCs strongly correlated with enhanced HIV uptake. Furthermore, viral uptake was dependent upon Siglec-1, as was demonstrated by the fact that blocking Siglec-1 with antibodies or reducing its expression with RNA interference strongly impaired HIV uptake.

These results suggested that Siglec-1 mediates uptake of HIV by mature DCs. In agreement with this idea, uptake of virus-like particles (which contain viral envelope gangliosides but lack HIV surface proteins), decreased when mature DCs were treated with antibodies that block Siglec-1, or when Siglec-1 expression was knocked down. Therefore, viral uptake depends on Siglec-1 binding to HIV envelope gangliosides.

If Siglec-1 controls DC viral uptake, it may also affect how efficiently HIV can be transferred from DCs to T cells. Mature DCs make intimate contact with myriad T cells, and it's been shown that HIV concentrates at sites of T-cell-DC contact. This promotes transmission of viruses to T cells, a process known as *trans*-infection. The group therefore examined whether blocking Siglec-1-mediated HIV uptake can inhibit *trans*-infection—and indeed, they found it does. Conversely, they observed that expressing Siglec-1 in laboratory cell lines that normally lack this protein rendered these cells capable of *trans*-infecting T cells.

Altogether, these data indicate that HIV can exploit Siglec-1 on mature DCs to promote both viral uptake and transmission to T cells. However, the authors remark that their results don't rule out a role for DC-SIGN in DC-HIV interactions; both Siglec1 and DC-SIGN are present on immature DCs, so both proteins probably contribute to HIV uptake and *trans*-infection by these cells. Nonetheless, mature DCs have a much higher capacity to capture HIV and *trans*-infect T cells than do immature DCs, and this activity is driven by Siglec-1, which highlights the importance of this interaction.

Izquierdo-Useros et al. note that Siglec-1 is a long protein with the potential to recognize a wide range of pathogens. Future work may therefore point the way toward therapeutic interventions against HIV and viruses that use similar infection strategies.

**Izquierdo-Useros N, Lorizate M, Puertas MC, Rodriguez-Plata MT, Zangger N, et al. (2012) Siglec-1 Is a Novel Dendritic Cell Receptor That Mediates HIV-1** ***Trans*** **-Infection Through Recognition of Viral Membrane Gangliosides. doi:10.1371/journal.pbio.1001448**

